# Antiangiogenic agents in the treatment of recurrent or newly diagnosed glioblastoma: Analysis of single-agent and combined modality approaches

**DOI:** 10.1186/1748-717X-6-2

**Published:** 2011-01-07

**Authors:** Kathryn Beal, Lauren E Abrey, Philip H Gutin

**Affiliations:** 1 Department of Radiation Oncology, Memorial Sloan-Kettering Cancer Center, 1275 York Avenue, New York, NY 10021, USA; 2 Department of Neurology, Memorial Sloan-Kettering Cancer Center, 1275 York Avenue, New York, NY 10021, USA; 3 Department of Neurosurgery, Memorial Sloan-Kettering Cancer Center, 1275 York Avenue, New York, NY 10021, USA

## Abstract

Surgical resection followed by radiotherapy and temozolomide in newly diagnosed glioblastoma can prolong survival, but it is not curative. For patients with disease progression after frontline therapy, there is no standard of care, although further surgery, chemotherapy, and radiotherapy may be used. Antiangiogenic therapies may be appropriate for treating glioblastomas because angiogenesis is critical to tumor growth. In a large, noncomparative phase II trial, bevacizumab was evaluated alone and with irinotecan in patients with recurrent glioblastoma; combination treatment was associated with an estimated 6-month progression-free survival (PFS) rate of 50.3%, a median overall survival of 8.9 months, and a response rate of 37.8%. Single-agent bevacizumab also exceeded the predetermined threshold of activity for salvage chemotherapy (6-month PFS rate, 15%), achieving a 6-month PFS rate of 42.6% (p < 0.0001). On the basis of these results and those from another phase II trial, the US Food and Drug Administration granted accelerated approval of single-agent bevacizumab for the treatment of glioblastoma that has progressed following prior therapy. Potential antiangiogenic agents-such as cilengitide and XL184-also show evidence of single-agent activity in recurrent glioblastoma. Moreover, the use of antiangiogenic agents with radiation at disease progression may improve the therapeutic ratio of single-modality approaches. Overall, these agents appear to be well tolerated, with adverse event profiles similar to those reported in studies of other solid tumors. Further research is needed to determine the role of antiangiogenic therapy in frontline treatment and to identify the optimal schedule and partnering agents for use in combination therapy.

## Introduction

The incidence rates of primary malignant brain and central nervous system (CNS) cancers have increased over the last 3 decades [1], reaching an estimated rate of 6.8 new cases per 100,000 persons in the United States [2]. Glioblastoma is the most common primary malignant brain tumor and accounts for the majority of diagnoses. On the basis of data collected between 1995 and 2006, glioblastoma has been associated with a particularly poor prognosis, with survival rates at 1 and 5 years equaling 33.7% and 4.5%, respectively [3]. The current standard of care for patients with newly diagnosed glioblastoma is surgical resection followed by fractionated external beam radiotherapy and systemic temozolomide [4], as supported by data from a randomized phase III trial, which demonstrated a significant improvement with the addition of temozolomide to radiotherapy in median overall survival (OS) from 12.1 months to 14.6 months [5]. Although this treatment can prolong survival, it is not curative. The vast majority of patients with glioblastoma experience recurrent disease, with a median time to recurrence of 7 months [6].

Currently, there is no standard treatment for patients with recurrent glioblastoma, although additional surgery, chemotherapy, and radiotherapy are used. An analysis of data from phase II clinical trials showed the limitations of conventional chemotherapy regimens, which were associated with a 6-month progression-free survival (PFS) rate of 15% and a median OS of 25 weeks in patients with recurrent disease [7]. More recent trials of single-agent temozolomide or irinotecan, also known as CPT-11, have demonstrated only slight increases in 6-month PFS, with the highest rate being 26% [8-10]. Recommended chemotherapeutic options for recurrent glioblastoma include temozolomide, nitrosourea, cyclophosphamide, platinum-based combination regimens, and procarbazine, lomustine, and vincristine combination therapy [4]. Moreover, in May 2009, the US Food and Drug Administration (FDA) granted accelerated approval of single-agent bevacizumab for the treatment of patients with glioblastoma that has progressed following prior therapy [11]. The National Comprehensive Cancer Network (NCCN) guidelines have subsequently been amended to include a recommendation for the use of bevacizumab, with or without chemotherapy (i.e., irinotecan, bischloroethylnitrosourea, or temozolomide), for progressive glioblastoma [4]. Enrollment in a clinical trial is considered standard practice at recurrence.

**Figure 1:**
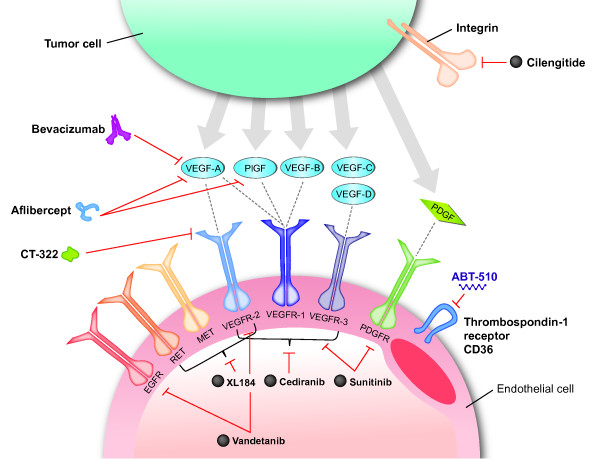
**Molecular targets of antiangiogenic agents in glioblastoma**. Cilengitide is a cyclic peptide that binds to and inhibits the activities of the alpha(v)beta(3) and alpha(v)beta(5) integrins. Bevacizumab is a humanized monoclonal immunoglobulin G1 antibody that binds to and inhibits VEGF-A. Aflibercept is a fusion protein that binds all isoforms of VEGF-A, as well as PlGF. Cediranib, sunitinib, vandetanib, XL184, and CT-322 are multireceptor tyrosine kinase inhibitors. ABT-510 is a nonapeptide that targets the thrombospondin-1 receptor CD36. Abbreviations: EGFR = epidermal growth factor receptor; PDGFR = platelet-derived growth factor receptor; PlGF = placental growth factor; VEGF-A = vascular endothelial growth factor A; VEGFR = vascular endothelial growth factor receptor.

Bevacizumab is a humanized monoclonal antibody that targets vascular endothelial growth factor (VEGF), an important mediator of angiogenesis that is essential for the tumorigenesis of glioblastoma. Antiangiogenic therapies may arrest tumor growth by mediating the regression of existing tumor vasculature and preventing regrowth over time [12,13]. As a result, bevacizumab and other antiangiogenic agents, including cediranib (AZD2171), aflibercept (VEGF Trap), XL184 and cilengitide (EMD 121974), are being evaluated for use in recurrent and newly diagnosed glioblastoma (Figure 1). This article reviews the available data from clinical trials of antiangiogenic agents in glioblastoma, either as single agents or in combination with chemotherapy and/or radiotherapy.

## Rationale For Using Antiangiogenic Therapies In The Treatment Of Glioblastoma

Glioblastomas are associated with a high degree of microvascular proliferation, and the extent of proliferation correlates with an increased risk of recurrence and poor survival [14]. VEGF-A (also known as "VEGF") is one of the most well-studied and potent vascular permeability factors, with an established role in pathologic angiogenesis [15]. Studies evaluating VEGF levels in plasma and tumor fluid from patients have shown that glioblastomas express relatively high levels of VEGF [16,17], and mean intracavitary levels of VEGF are significantly increased in patients with recurrent glioblastoma relative to those with nonrecurrent disease [16]. Moreover, there is a direct correlation between VEGF overexpression and poor prognosis in this tumor histology [18].

**Figure 2:**
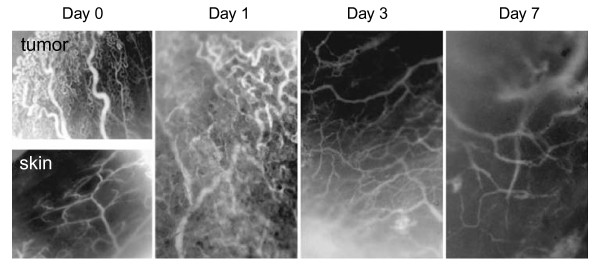
**Intravital microscopy images showing the effect of a single dose of bevacizumab on the intratumoral vascular phenotype of an orthotopic neuroblastoma (NB-1691 xenograft) model**. Images were obtained on days 0, 1, 3, and 7. Original magnification was ×40. Vasculature of normal skin is also shown [19]. Reprinted with permission from Dickson PV, Hamner JB, Sims TL, *et al*. Bevacizumab-induced transient remodeling of the vasculature in neuroblastoma xenografts results in improved delivery and efficacy of systemically administered chemotherapy. *Clin Cancer Res *2007;13:3942-3950; Figure 2.

Preclinical studies have provided evidence that the inhibition of the VEGF ligand can modulate tumor vasculature. In a study using neuroblastoma xenografts, Dickson and colleagues demonstrated that treatment with bevacizumab led to reductions in microvessel density and improvement in the function of intratumoral blood vessels (Figure 2), facilitating the penetration of subsequent chemotherapy [19]. In another glioblastoma model, bevacizumab suppressed both the proangiogenic effects of stem cell-like glioma cells (SCLGCs) *in vitro *and the growth of SCLGC-derived glioblastoma xenografts *in vivo *[20]. Data also suggest an association between other proangiogenic factors, such as the angiopoietins, neuropilin-1, and delta-like ligands, and the survival and/or proliferation of tumor cells [21-23]. Collectively, these results highlight the importance of VEGF and the related signal transduction pathways as therapeutic targets in glioblastoma and provide the rationale for evaluating antiangiogenic agents in clinical trials.

**Figure 3:**
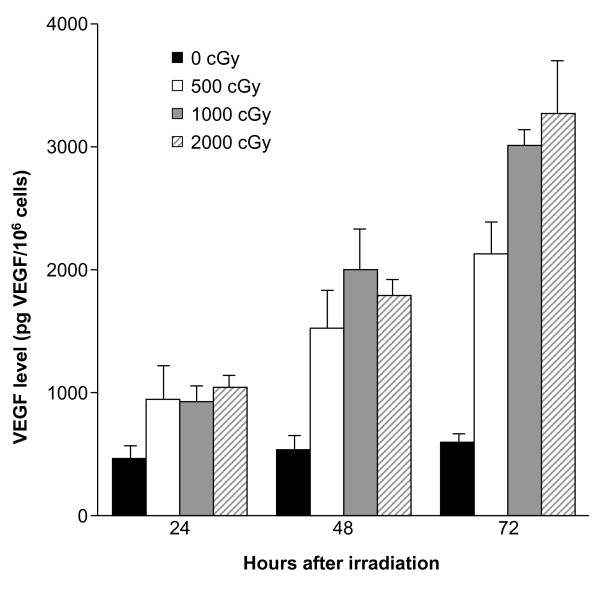
**Dose-dependent effect of radiation on VEGF protein expression**. VEGF protein levels in LLC-conditioned medium are shown after radiation exposure. LLCs were plated in six-well plates at 25% confluence, allowed to attach overnight, and then irradiated with 0, 5, 10, or 20 Gy. Conditioned medium was collected every 24 hours and VEGF levels were normalized to cell number. Data are presented as mean plus standard error [70]. Abbreviations: LLC = Lewis lung carcinoma; VEGF = vascular endothelial growth factor. Reprinted with permission from Gorski DH, Beckett MA, Jaskowiak NT, *et al*. Blockage of the vascular endothelial growth factor stress response increases the antitumor effects of ionizing radiation. *Cancer Res *1999;59:3374-3378; Figure 1B.

## Clinical Experience With Antiangiogenic Agents In Glioblastoma

### Antiangiogenic agents with chemotherapy for recurrent glioblastoma

In the initial investigation in patients with recurrent glioblastoma, bevacizumab was evaluated in combination with concomitant irinotecan [24]. This combination was supported by the activity of bevacizumab with irinotecan-containing regimens in patients with metastatic colorectal cancer [25], by the relative lack of single-agent activity of thalidomide in recurrent glioblastoma [26], and by preclinical evidence, suggesting that antiangiogenic agents enhance intratumoral chemotherapy delivery [19,27]. Additionally, antiangiogenic agents may supplement the effect of chemotherapy by inhibiting the activity of a population of SCLGCs that is not as well differentiated (i.e., chemotherapy-resistant) [20]. The existence of these cells may partially explain tumor resistance to radiotherapy and chemotherapy, and could contribute to the recurrence of glioblastoma.

### Use of bevacizumab with chemotherapy

Data from prospective and retrospective studies indicate that regimens combining bevacizumab and chemotherapy produce superior outcomes relative to those with conventional chemotherapy in patients with recurrent glioblastoma [7]. In the first prospectively designed, phase II trial, patients with recurrent glioblastoma received bevacizumab plus irinotecan in one of two treatment cohorts: the first cohort (n = 23) received bevacizumab 10 mg/kg plus irinotecan q2w in a 6-week cycle, and a second cohort (n = 12) received bevacizumab 15 mg/kg q3w with irinotecan on days 1, 8, 22, and 29 of a 6-week cycle [28,29]. In both cohorts, irinotecan was administered at 340 mg/m^2 ^to 350 mg/m^2 ^in patients on enzyme-inducing antiepileptic drugs (EIAEDs) and at 125 mg/m^2 ^in those not receiving EIAEDs. The 6-month PFS rate among all 35 patients was 46%, the 6-month OS rate was 77%, and the median OS was 42 weeks [29]. In addition, the overall response rate (ORR) was high (57%). Recently, the 4-year survival rate in this trial was reported to be 11% [30] (Table 1). The toxicity of the combination of bevacizumab and irinotecan was considered to be significant but acceptable, considering the poor prognosis of the population [29]. Eleven (31%) of the 35 patients discontinued treatment because of adverse events, which included thromboembolic complications (n = 4), grade 2 proteinuria (n = 2), and grade 2 fatigue (n = 4); one patient experienced a CNS hemorrhage.

**Table 1:** Efficacy outcomes with antiangiogenic agents in recurrent glioblastoma.

Reference, (n^a^)	Treatment regimen	Response rate (%)^b^			Progression-free survival	
		CR	PR	SD	Median	At 6 months (%)
**Bevacizumab**						
Vredenburgh [28], (n = 23 of 32)^d^	BV + irinotecan	4	57	35	20 weeks	30
Vredenburgh [29], (N = 35)	BV + irinotecan	57		N/A	24 weeks	46
Narayana [33], (n = 37 of 61)^e^	BV + irinotecan or carboplatin	13	60	21	5 months	N/A
Friedman [31], Cloughesy [32], (N = 167)	BV alone (n = 85) BV + irinotecan (n = 82)	28 38		N/A N/A	4.2 months 5.6 months	43 50
Reardon [38], (n = 27 of 59)^d^	BV + etoposide	4	19	70	18 weeks	44
Kreisl [49], (N = 48)	BV → BV + irinotecan	71 (Levin criteria); 35 (MacDonald criteria)		N/A	16 weeks	29
Gutin [92], (n = 20 of 25)^d^	BV + hypofractionated stereotactic irradiation	50		N/A	7.3 months	65
**Aflibercept**						
De Groot [53], (n = 32 of 48)^d^	Aflibercept alone	0	30	52	N/A	N/A
**Cediranib**						
Batchelor [112], (N = 31)	Cediranib alone	57 (volumetric criteria); 27 (MacDonald criteria)		N/A	117 days	26
**Cilengitide**						
Reardon [50] (N = 81)	Cilengitide alone (2000 mg/d [n = 40] or 500 mg/d [n = 41])	0	9	N/A	2000 mg/d, 8.1 weeks^c^ 500 mg/d, 7.9 weeks^c^	2000 mg/d, 15 500 mg/d, 10
**CT-322**						
Schiff [113], (n = 51)	CT-322 alone (n = 33) CT-322 + irinotecan (n = 18)	1 (3) 0	1 (3) 0	N/A N/A	N/A N/A	23 48
**XL184**						
Wen [54], (n = 105)	XL184 175 mg qd XL 184 125 mg qd	AAT-naive (n = 34), 21 AAT-pretreated (n = 12), 8 AAT-naive (n = 37), 30 AAT-pretreated (n = 22), 0		N/A	AAT-naive, 16 weeks AAT-naive, 16 weeks AAT-pretreated, 7.9 weeks	AAT-naive, 10 AAT-naive, 25 AAT-pretreated, 0

Abbreviations: AAT = antiangiogenic therapy; BV = bevacizumab; CR = complete response; N/A = not available; PR = partial response; RT = radiotherapy; SD = stable disease.

^a^Number of patients with glioblastoma, where available.

^b^In evaluable patients.

^c^Time to progression.

^d^Efficacy outcomes are reported for patients with glioblastoma only.

^e^Efficacy outcomes are reported for all patients.

More recently, Friedman and colleagues investigated the use of bevacizumab with or without irinotecan in a randomized noncomparative phase II trial of 167 patients with recurrent glioblastoma-the BRAIN study [31,32]. In this trial, patients were randomized to bevacizumab 10 mg/kg q2w alone (n = 85) or in combination with irinotecan (n = 82). For patients treated with bevacizumab and irinotecan, the estimated 6-month PFS rate was 50.3%, the median OS was 8.9 months, and the ORR was 37.8% at the 6-month follow-up. At 27 months of follow-up, the 12-, 18-, 24-, and 30-month survival rates were 38%, 18%, 17%, and 16%, respectively. In the safety population for the combination arm (n = 79), the most common grade ≥ 3 adverse events were convulsion (13.9%), neutropenia (8.9%), and fatigue (8.9%). Adverse events led to treatment discontinuation for 14 (17.7%) patients. Adverse events associated with bevacizumab included grade ≥ 3 arterial thromboembolism (2.5%), grade ≥ 3 wound-healing complications (1.3%), grade ≥ 3 venous thromboembolism (10.1%), grade 3 gastrointestinal perforation (2.5%), serious reversible posterior leukoencephalopathy syndrome (1.3%), and intracranial hemorrhage (3.8%). In addition, there was one death associated with convulsion in patients treated with bevacizumab and irinotecan.

Data from additional phase II studies, retrospective analyses, and case series of consecutive patients have provided further support for the activity of bevacizumab with chemotherapy in patients with recurrent glioblastoma [33-39]. In these studies, 6-month PFS rates have ranged from 6.7% to 64% in patients with recurrent glioblastoma. In general, bevacizumab was shown to be well tolerated in both prospective and retrospective studies, and no unexpected treatment-related adverse events were reported (Table 2). Reported events were typical of those associated with bevacizumab in the treatment of other tumor types. For example, hypertension and proteinuria have been reported as the most frequently occurring treatment-related adverse events in studies of bevacizumab therapy in other solid tumors [11,25,40]. The incidence of thromboembolic complications in patients with recurrent glioblastoma receiving bevacizumab plus chemotherapy ranged from 11.4% to 12.7% in the two prospective studies [28,29,32]. The relation of bevacizumab to these events, however, is unclear because patients with malignant gliomas are already at an increased risk for symptomatic venous thromboembolism. In a retrospective study of 9489 cases of malignant glioma, the 2-year cumulative incidence of venous thromboembolism was relatively high at 7.5% (n = 715 cases) [41]. Furthermore, a diagnosis of glioblastoma was identified as a specific risk factor for venous thromboembolism (hazard ratio [HR] = 1.7; 95% confidence interval [CI], 1.4-2.1). Overall, the safety profile of bevacizumab with chemotherapy has been within acceptable limits, without any indications of additive toxicities.

**Table 2:** Safety profile of antiangiogenic agents for recurrent glioblastoma.

**Reference, (n**^**a**^**)**	Treatment regimen	Patients discontinuing because of an adverse event, n (%)	Select grade 3 or 4 adverse events, **n (%)**^**b**^	Intracranial hemorrhage (any grade), n (%)	Thromboembolic events (any grade), n (%)	Treatment- related deaths, n (%)
**Bevacizumab-containing regimens**						
Vredenburgh [28], (n = 23 of 32)^c^	BV + irinotecan	9 (28.1)	N/A	0	4 (12.5)	2 (6.3)
Vredenburgh [29], (N = 35)	BV + irinotecan	11 (31.4)	N/A	1 (2.9)	4 (11.4)	N/A
Narayana [33], (n = 37 of 61)^c^	BV + irinotecan or carboplatin	16 (26.2)	Bone marrow toxicity, 6 (9.8)	6 (9.8)	6 (9.8)	0
Friedman [31], Cloughesy [32], (N = 167)	BV alone (n = 84) BV + irinotecan (n = 79)	4 (4.8) 14 (17.7)	All, 43 (51.2) Hypertension, 9 (10.7) Wound-healing complications, 2 (2.4) Proteinuria, 1 (1.2) All, 56 (70.9) Hypertension, 3 (3.8) Wound-healing complications, 1 (1.3) Proteinuria, 3 (3.8) GI perforation, 2 (2.5)	3 (3.6) 3 (3.8)	ATE, 4 (4.8) VTE, 3 (3.6) ATE, 3 (3.8) VTE, 9 (11.4)	2 (2.4) 1 (1.3)
Reardon [38], (n = 27 of 59)^c^	BV + etoposide	7 (11.9)	Neutropenia, 14 (23.7) Infection, 5 (8.5) Hypertension, 2 (3.4)	CNS hemorrhage, 2 (3.4)	7 (11.9)	1 (1.7)
Kreisl [49], (N = 48)	BV → BV + irinotecan	6 (12.5)	Hypertension, 2 (4.2) Hypophosphatemia, 2 (4.2) Bowel perforation, 1 (2.1)	0	6 (12.5)	N/A
						
Gutin [92], (n = 20 of 25)^c^	BV + hypofractionated stereotactic irradiation	3 (12)	Lymphopenia, 9 (36) Hyponatremia, 6 (24) Bowel perforation, 1 (4) Wound-healing complication, 1 (4%) GI bleeding, 1 (4%)	1 (4)	N/A	N/A

**Aflibercept**						
De Groot [53], (n = 32 of 48)^c^	Aflibercept alone	12 (25)	CNS ischemia, 1 (2.1) Systemic hemorrhage, 1 (2.1)	N/A	N/A	N/A

**Cediranib**						
Batchelor [112], (N = 31)	Cediranib alone	2 (6.5)	Fatigue, 6 (19.4) ALT, 5 (16.1) Hypertension, 4 (12.9)	N/A	1 (3.2)	0

**Cilengitide**						
Reardon [50], Fink [51], (N = 81)	Cilengitide alone (2000 mg/d [n = 40] or 500 mg/d [n = 41])	N/A	Convulsion, 2 (2.5) Lymphopenia, 7 (8.6) Neutropenia, 1 (1.2)	1 (1.2)	N/A	5 (6.2)

**CT-322**						
Schiff [113], (n = 51)	CT-322 ± irinotecan	13 (25.5)	Neutropenia, 4 (7.8) Hypertension, 3 (5.9)	CNS hemorrhage, 1 (2.0)	N/A	1 (2.0)

**XL184**						
Wen [54], (n = 153)	XL184 (175 mg qd [n = 46] or 125 mg qd [n = 107])	18 (11.8)	Fatigue, 31 (20.3) Hypertension, 8 (5.2) GI perforation, 3 (2.0) Wound-healing complications, 2 (1.3)	3 (2.0; grade 3/4)	17 (11.1)	N/A

**Abbreviations: **ALT = alanine transaminase; ATE = arterial thromboembolic event; BV = bevacizumab; CNS = central nervous system; GI = gastrointestinal; N/A = not available; VTE = venous thromboembolic event.

^a^Number of patients with glioblastoma, where available.

^b^Intracranial hemorrhage and thromboembolic events are reported separately.

^c^Safety outcomes are reported for all patients.

### Other antiangiogenic therapies used with chemotherapy for recurrent glioblastoma

Clinical trials have also evaluated the safety and efficacy of other antiangiogenics, specifically thalidomide and vatalanib, in combination with chemotherapy agents. In phase II trials of patients with recurrent glioblastoma, thalidomide-containing regimens produced 6-month PFS rates between 23% and 27% and objective response rates between 6% and 24% [42-45]. Although the findings of two of these studies suggested that combination therapy was more active than either thalidomide or the chemotherapy partner alone, the benefit-to-risk ratio of thalidomide-containing therapy has not been clearly established, particularly when considering that certain combinations are complicated by significant adverse events (e.g., neutropenia and thromboembolism). A phase I/II trial of vatalanib plus temozolomide (n = 37) or lomustine (n = 23) provided evidence of activity in patients with recurrent glioblastoma-patients receiving vatalanib and temozolomide had a median time to progression of 16.1 weeks and a partial response rate of 9% across all dose groups [46]. However, vatalanib has since been discontinued from further investigation in patients with glioblastoma.

### Single-agent activity of antiangiogenic therapies in recurrent glioblastoma

As data from trials of antiangiogenic agents and chemotherapy in the recurrent setting began to emerge, questions arose about the relative contribution of concomitant cytotoxic therapy in these regimens. Single-agent antiangiogenic strategies were effective in other solid tumors, including renal cell carcinoma and ovarian cancer [40,47,48]. Thus, clinical trials were initiated to investigate whether single-agent approaches were appropriate in patients with recurrent glioblastoma, anticipating that they might provide antitumor control while minimizing toxicity.

#### Single-agent bevacizumab

The approval of single-agent bevacizumab treatment for patients with recurrent glioblastoma was based on an improvement in objective response rates in two phase II studies [31,49]. In a study by Kreisl and colleagues, 48 patients with heavily pretreated glioblastoma (median of two prior chemotherapy regimens) received bevacizumab 10 mg/kg q2w until disease progression [49]. At progression, patients received bevacizumab plus irinotecan. During the monotherapy phase of the study, the median PFS was 16 weeks (95% CI, 12-26 weeks), the 6-month PFS rate was 29% (95% CI, 18%-48%), and the ORR was 35% (one complete response). When response assessment criteria were based on both World Health Organization (WHO) radiographic criteria and on stable or decreasing corticosteroid use, the objective response rate was 19.6% (95% CI, 10.9%-31.3%) [11]. The median OS was 31 weeks (95% CI, 21-54 weeks), and the 6-month OS was 57% (95% CI, 44%-75%). Single-agent bevacizumab was well tolerated; the most frequently observed treatment-related adverse events were grade 3 or 4 thromboembolic events (12.5%), grade 2 or 3 hypertension (12.5%), grade 2 or 3 hypophosphatemia (6%), and grade 2 or 3 thrombocytopenia (6%). Of the six patients (12.5%) who experienced a thromboembolic event, three had pulmonary emboli and one had a cerebral vascular event. Thromboembolic events in five patients and one instance of bowel perforation in another led to the removal of six patients (12.5%) from the study. No instances of intracranial hemorrhage were reported.

The safety and efficacy of single-agent bevacizumab was further substantiated by a large, randomized, noncomparative phase II study (BRAIN) in which patients with glioblastoma in first or second relapse were randomized to bevacizumab alone or in combination with irinotecan [31,32]. Outcomes for patients treated with both bevacizumab and irinotecan in the BRAIN study have been described earlier. Patients who received bevacizumab monotherapy (n = 85) had a 6-month PFS rate of 42.6% (95% CI, 29.6%-55.5%), an ORR of 28.2% (one complete response), and a median OS of 9.3 months. Responses, categorized both by WHO radiographic criteria and by stable or decreasing corticosteroid use, were seen in 25.9% (95% CI, 17.0%-36.1%) of patients [11]. As in the combination arm, the 6-month PFS rate in the monotherapy arm surpassed the 15% rate assumed for salvage chemotherapy and single-agent irinotecan (p < 0.0001). The 12-, 18-, 24-, and 30-month OS rates were 38%, 24%, 16%, and 11%, respectively. No unexpected adverse events were reported, and there was a low incidence of intracranial hemorrhage. Forty-three (51.2%) patients eligible for the safety analysis (n = 84) had grade ≥ 3 adverse events, including hypertension (10.7%), venous thromboembolism (3.6%), wound-healing complications (2.4%), and arterial thromboembolism (3.6%). Three (3.4%) patients who received single-agent bevacizumab experienced intracranial hemorrhage; all of these events were grade ≤2. Two patients died as a result of an adverse event (neutropenia infection and pulmonary embolism, respectively), and four patients (4.8%) discontinued bevacizumab treatment because of adverse events.

Although the randomized design of the trial was intended only to prevent bias in treatment assignment and not to compare outcomes in the two treatment groups, it is notable that bevacizumab monotherapy was associated with a lower rate of grade ≥ 3 adverse events (51.2% vs 70.9%) than the combination of bevacizumab and irinotecan [32]. Furthermore, in the two studies evaluating bevacizumab monotherapy [31,49], the rate of treatment discontinuation owing to adverse events was relatively low (4.8% and 12.5%, respectively) compared with discontinuation rates in the bevacizumab-plus-irinotecan arms of the Friedman and Vredenburgh studies (17.7% and 31%, respectively) [29,31]. This suggests that the rate of certain adverse events, such as infection, may be reduced or even eliminated by the omission of chemotherapy.

#### Single-agent data with other antiangiogenic agents

A number of other antiangiogenic therapies have been studied or are being studied as single-agent therapy in patients with recurrent glioblastoma, including cilengitide, aflibercept, XL184, cediranib, sunitinib, and CT-322 (see Tables 1 and 2). Long-term follow-up results with the integrin inhibitor cilengitide have recently been reported from a phase II trial in 81 patients with recurrent glioblastoma, in which cilengitide 500 mg (n = 41) or 2000 mg (n = 40) was given twice weekly [50,51]. Median OS was 9.9 months in the 2000-mg arm compared with 6.5 months in the 500-mg arm. OS rates were consistently greater with the 2000-mg dose of cilengitide (37%, 23%, 15%, and 10% at 12, 24, 36, and 48 months, respectively) compared with the 500-mg dose (22%, 12%, 5%, and 2%, at 12, 24, 36, and 48 months, respectively) (HR = 0.635). Cilengitide was well tolerated, with no significant reproducible toxicities in the dose groups. For the 15 patients who received cilengitide for more than 6 months, treatment-related adverse events tended to occur within 6 months of receiving the first dose of cilengitide; the most common treatment-related adverse event was fatigue (n = 3), and the most common grade 3 or 4 serious adverse event was convulsion (n = 2). Only two patients reported serious adverse events from 6 months up to 4.5 years from the first cilengitide dose (headache and memory impairment). The investigators concluded that cilengitide monotherapy was well tolerated and feasible for >4 years of therapy, with long-term survival rates being consistently greater with the 2000-mg dose.

Aflibercept is a recombinantly produced fusion protein that binds both VEGF and placental growth factor and has been shown to suppress the growth of glioblastoma xenografts in murine models [52]. In NABTC 0601, an ongoing phase II study, patients with temozolomide-resistant glioblastoma or anaplastic glioma at first relapse receive aflibercept 4 mg/kg q2w [53]. Preliminary efficacy data in 27 patients with glioblastoma revealed an ORR of 30%. Aflibercept showed moderate tolerability-the rate of treatment discontinuation among all 48 enrolled patients was 25%. Eighteen treatment-related, grade 3 adverse events were reported. Mature data will provide a better indication of the activity of single-agent aflibercept in the recurrent setting.

Recently, interim results from a phase II study of XL184, an oral inhibitor of multiple receptor tyrosine kinases that includes VEGF receptor 2, in previously treated progressive glioblastoma have been reported [54]. In the cohort treated with XL184 175 mg (n = 46), the ORRs were 8% (1/12) and 21% (7/34) in patients with and without previous exposure to antiangiogenic treatment, respectively. While none of the 22 patients previously treated with antiangiogenic therapy responded to XL184 125 mg, the ORR in patients with antiangiogenic-naive disease was 30% (11/37) with the 125-mg dose. The median PFS in both antiangiogenic-naive cohorts was 16 weeks. In total, 61% (46/76) of patients on corticosteroids at baseline had a reduction in corticosteroid dose of at least 50%. Common grade 3 or 4 adverse events among all 153 evaluable patients included fatigue (20%), transaminase elevation (12%), and thromboembolic events (10%). The investigators concluded that XL184 demonstrates encouraging clinical activity in patients with progressive glioblastoma and that the 125-mg dose of XL184 demonstrates improved tolerability compared with the 175-mg dose.

### Continued use of antiangiogenic agents after progression

In the event of progression following treatment with an antiangiogenic agent, patients with glioblastoma have very few therapeutic options. For example, in a prospective study by Kreisl and colleagues, a cohort of 19 patients was subsequently treated with bevacizumab plus irinotecan after progression on bevacizumab monotherapy [49]. None of these patients responded to therapy, and the median PFS was 30 days. In another prospective phase II study of patients with recurrent malignant gliomas treated with daily temozolomide, it was found that patients with prior exposure to bevacizumab fared worse than patients without bevacizumab exposure (6-month PFS rate of 14% vs 36%, p = 0.12; median OS of 4 vs 18 months, p = 0.005) [55]. Retrospective reviews of patients with glioblastoma treated either with a bevacizumab-containing regimen or bevacizumab alone have also reported that these patients have limited response to a second treatment, regardless of whether it contains bevacizumab [36,56-59]. One hypothesis for the lack of response after antiangiogenic treatment is that an alteration of the tumor phenotype results in a highly infiltrative compartment that is angiogenic-independent. Further studies are warranted to identify new therapeutic targets and novel agents that could treat patients who have relapsed following antiangiogenic therapy.

One of the concerns with the administration of antiangiogenic agents is the apparent potential for infiltrative or invasive tumor growth upon disease progression [33,35,36,60-62]. Recent reports, however, indicate that antiangiogenic treatments may not significantly alter patterns of relapse in glioblastoma. For example, in a study of distant spread in 44 matched pairs of patients with recurrent glioblastoma treated with or without bevacizumab-containing regimens, distant recurrences were later observed in 22% (10/44) of bevacizumab-treated patients compared with 18% (8/44) of non-bevacizumab-treated patients on T_1_-weighted magnetic resonance imaging (MRI) scans, and in 25% (11/44) of bevacizumab-treated patients compared with 18% (8/44) of non-bevacizumab-treated patients on fluid attenuation inversion recovery (FLAIR) MRI sequences (p > 0.05). This proportion of distant recurrences was in line with previous reports, without significant differences between bevacizumab and non-bevacizumab-containing treatments [63]. Moreover, a subanalysis of the BRAIN study, in which patient MRI scans were compared at baseline (prior to bevacizumab treatment) and at the time of progression, showed that the majority of patients (55/67 in the bevacizumab-alone group) had no shift in the pattern of progression. A shift from local to diffuse disease was seen in 16% (11/67) of patients in the bevacizumab-alone group [64]. Other investigators have likewise concluded on the basis of retrospective analyses of radiographic patterns of relapse that the majority of disease patterns with glioblastoma are local at diagnosis and remain so after recurrence and treatment with bevacizumab, and that the rate of nonlocal disease (diffuse, distant, or multifocal) does not appear to increase with the use of antiangiogenic agents [65-67]. Reports have also differed regarding the impact of the pattern of radiographic recurrence on survival outcomes [36,58,64,67]. In cases in which an infiltrative phenotype is observed at diagnosis, it is possible that antiangiogenic therapy in combination with another agent that targets tumor invasion, such as dasatinib [68], may be an effective therapeutic strategy.

### Antiangiogenic agents in combination with radiation

Increased understanding of molecular mechanisms in the tumorigenesis of glioblastomas has led to the evaluation of targeted agents as potential radiosensitizers [69,70]. Preclinical models have shown that VEGF is upregulated in response to radiation, and these elevations may contribute to the protection of tumor blood vessels from radiation-mediated cytotoxicity [70,71]. The administration of antiangiogenic agents with radiotherapy may counteract VEGF-mediated radioresistance, thereby sensitizing tumors and associated vasculature to the ionizing effects of radiation (Figure 3) [69,70,72]. As an underlying mechanism, the ability of antiangiogenic agents to lower tumor interstitial fluid pressure and improve vascular function and tumor oxygenation may promote enhanced responsiveness to radiotherapy [73,74]. Preclinical studies have also demonstrated that antiangiogenic agents uniquely target the radioresistant and highly tumorigenic cancer stem cell niche [20,75]. Finally, the success of initial clinical investigations of bevacizumab with chemoradiation in patients with solid tumors also supports the possible synergies of combined modality therapy [76,77].

### Efficacy of antiangiogenic agents and chemoradiation

The efficacy and safety of bevacizumab with chemotherapy and radiotherapy have been assessed in clinical studies for the treatment of both recurrent and newly diagnosed glioblastoma [78,79]. In the frontline setting, the use of bevacizumab plus radiotherapy and temozolomide has been described in two reports. In a phase II pilot study, 10 patients with glioblastoma underwent surgery followed by radiotherapy (30 fractions of 2 Gy per fraction) with bevacizumab 10 mg/kg q2w plus concomitant temozolomide 75 mg/m^2 ^[78]. Temozolomide therapy was continued until disease progression or for a maximum of 24 cycles, while bevacizumab therapy continued every 2 weeks until progression. At the time of reporting, the median PFS was >8.8 months, but it was too early to establish the median OS. The most commonly occurring, possibly treatment-related adverse events were fatigue, myelotoxicity, wound-healing complications, and venous thromboembolic events. The only unexpected toxicity was the development of presumed radiation-induced optic neuropathy in one patient. The study investigators noted, however, that the observed toxicities were at an acceptable level to continue enrollment toward a target of 70 patients.

In a subsequent feasibility study in a consecutive series of patients, Narayana and colleagues reported outcomes from 15 patients with high-grade glioma, including 12 patients with glioblastoma, who underwent surgery followed by radiotherapy (59.4 Gy over 6.5 weeks) [79]. Bevacizumab 10 mg/kg was administered on days 14 and 28 along with concomitant temozolomide 75 mg/m^2 ^daily during radiotherapy. After the completion of radiotherapy, treatment with bevacizumab and temozolomide continued for 12 cycles. At a median follow-up of 12 months (range, 5-21 months), the PFS rate was 59.3% and the OS rate was 86.7%. Nonhematologic toxicities were reported in three patients (20%), and grade 3 or 4 hematologic toxicities were reported in another three patients (20%) [79]. No intracerebral hemorrhage or treatment-related deaths occurred during the study. Several ongoing clinical trials have also recently reported interim data on the use of bevacizumab with radiotherapy and either temozolomide or irinotecan in patients with previously untreated glioblastoma [80-86]. In two of the trials with longer follow-up, the addition of bevacizumab with or without irinotecan to standard radiotherapy and temozolomide was shown to provide significant benefit in PFS relative to historic controls [80,82]. In one trial having a minimum follow-up of 18 months, the regimen incorporating bevacizumab and irinotecan was associated with a median PFS that was approximately double that seen with standard therapy in patients with newly diagnosed glioblastoma (14 vs 6.9 months, respectively) [82]. In both trials, the incorporation of bevacizumab into standard frontline regimens was considered to be tolerable [80,82]. Large phase III studies evaluating bevacizumab-containing regimens in patients with newly diagnosed glioblastoma have recently begun enrolling patients, including a global-based study (AVAglio [NCT00943826]) [87] and a US-based study (RTOG-0825 [NCT00884741], which is sponsored by the Radiation Therapy Oncology Group).

Results from a phase I/II trial of cilengitide in combination with temozolomide and radiotherapy in patients with newly diagnosed glioblastoma have also demonstrated promising efficacy [88]. After tumor resection, 52 patients received standard radiotherapy (2 Gy × 30 fractions) and temozolomide 75 mg/m^2^, with cilengitide 500 mg twice weekly started 1 week before chemoradiation and given throughout the duration of chemotherapy or until progression. The 6-and 12-month PFS rates were 69% and 33%, respectively; the median PFS was 8.0 months. The 12- and 24-month OS rates were 68% and 35%, respectively, with a median OS of 16.1 months. The authors reported that PFS and OS in patients with O(6)-methylguanine-DNA methyltransferase (MGMT) promoter methylation (13.4 and 23.3 months, respectively) were longer than those in patients without MGMT promoter methylation (3.4 and 13.1 months, respectively). Seven patients (14%) discontinued treatment for adverse events that were possibly treatment-related. The regimen was found to be well tolerated, with no additional toxicities [88].

Early phase studies have evaluated additional antiangiogenic agents, such as vatalanib, vandetanib, and ABT-510, in combination with temozolomide and radiotherapy for the treatment of patients with newly diagnosed glioblastoma [89-91]. These trials provide further evidence for the feasibility of combining these treatment modalities in the frontline setting.

Recent studies have also reported on the feasibility of using bevacizumab with radiotherapy in patients with recurrent malignant gliomas [92,93]. One of these studies reported outcomes in 25 patients (20 patients with glioblastoma and five patients with anaplastic glioma) who received bevacizumab 10 mg/kg q2w until tumor progression, along with hypofractionated stereotactic radiotherapy (30 Gy total as 6 Gy × 5 fractions) after the first cycle of bevacizumab therapy [92]. In the glioblastoma cohort, the regimen was associated with a 6-month PFS rate of 65% (95% CI, 40%-82%) and a median PFS of 7.3 months (95% CI, 4.4-8.9 months). The median OS was 12.5 months (95% CI, 6.9-22.8 months), the 1-year survival was 54%, and the ORR was 50%. The overall toxicity of the regimen was comparable to that in other clinical trials of bevacizumab in glioblastoma [28,29,31,78]. Three patients in the overall population experienced a grade 4 adverse event-bowel perforation, wound-healing complication, and gastrointestinal bleeding. Other nonhematologic and hematologic toxicities were transient. No significant adverse events appeared to be attributable to the interaction of bevacizumab with radiation, with the exception of a single instance of wound dehiscence; radiation necrosis was not observed in this previously irradiated population. Overall, the high 6-month PFS rate and improved therapeutic ratio of this combination suggest that it should be investigated in larger trials of patients with recurrent disease and supports ongoing trials of bevacizumab with radiochemotherapy in patients with newly diagnosed glioma.

### Other considerations with antiangiogenic therapies

The role of antiangiogenic therapy also requires further evaluation of its potential use in glioblastoma-related conditions. One example is pseudoprogression, which may be visualized on brain scans in patients who have received chemoradiotherapy and temozolomide, resulting from increased cerebral edema. In clinical studies, both bevacizumab and cediranib have shown activity in reducing the need for steroid therapy to treat tumor-associated cerebral edema [31,94]. Therefore, these agents may be useful in cases in which pseudoprogression is suspected, as well as in patients with large, inoperable glioblastomas who are dependent on steroid therapy.

Antiangiogenic treatment has also been proposed for the management of radiation necrosis, a process in which endothelial cell dysfunction leads to tissue hypoxia and necrosis, with the concomitant release of vasoactive compounds [95]. In a small randomized double-blind study, Levin and colleagues reported outcomes in 14 patients who received either placebo or bevacizumab for radiographically-proven or biopsy-proven CNS necrosis. All of the bevacizumab-treated patients (5/5 randomized patients; 7/7 crossover patients), but none of the placebo-treated patients (n = 7), showed improvement in neurologic symptoms or signs and had a reduction in the volume of necrosis on T_2_-weighted FLAIR (average reduction of 59% in randomized patients) and T_1_-weighted gadolinium-contrast MRI (average reduction of 63% in randomized patients) [96]. Similar radiographic responses, along with improved or stable clinical outcomes, were also achieved with bevacizumab treatment in a retrospective analysis of eight patients with documented radiation necrosis [95], as well as a case series of six patients with biopsy-proven radiation necrosis [97].

In addition to its role in the treatment of glioblastoma, bevacizumab has also been evaluated in other high-grade gliomas. Results from phase II studies and retrospective reviews of bevacizumab for the treatment of anaplastic gliomas have been encouraging. In a phase II study of 33 patients with recurrent grade 3 malignant gliomas (anaplastic astrocytoma, anaplastic oligodendroglioma, and anaplastic oligoastrocytoma), Desjardins and colleagues found the use of bevacizumab and irinotecan to be active (6-month PFS = 55%; 6-month OS = 79%; ORR = 61%) and to have acceptable toxicity, with infrequent significant adverse events [98]. In a more recent study of 31 patients with recurrent anaplastic glioma, single-agent bevacizumab was associated with a median PFS of 3.7 months, a median OS of 12.4 months, reduced steroid requirements (a 40% reduction, on average, in steroid dose), and improved neurologic symptoms [99]. The activity and safety of single-agent bevacizumab have also been described in retrospective studies of patients with recurrent alkylator-refractory anaplastic oligodendroglioma and anaplastic astrocytoma [100,101]. The NCCN guidelines now include the use of bevacizumab with or without chemotherapy as a treatment option for recurrent anaplastic gliomas [4].

Another consideration is the impact of antiangiogenic agents on radiographic evaluations of treatment response in malignant gliomas. Some investigators argue that it is challenging to determine disease progression and tumor response to antiangiogenic therapy because of the effect of these agents on vascular permeability, which results in diminished contrast enhancement on computed tomography or MRI scans [102-104]. Because the current standard response criteria (MacDonald criteria) are based on contrast enhancement MRI, there is some debate as to whether these criteria are still adequate in the era of antiangiogenic agents. Proposals for new treatment response assessment criteria have been presented by various authors and also by the Response Assessment in Neuro-Oncology Working Group, and include taking into account T_2_/FLAIR (non-contrast enhancing) imaging, favoring the use of Levin criteria, or changing the criterion of response by cross-sectional area of enhancement measurement (e.g., a > 25% decrease vs ≥ 50% decrease) [35,99,105]. Additional imaging techniques and analyses for the assessment or predictors of antiangiogenic treatment response that have been proposed for additional investigation include FLAIR MRI, dynamic contrast-enhanced MRI, diffusion-weighted MRI, pretreatment apparent diffusion coefficient histogram analysis, and perfusion imaging or dynamic susceptibility contrast MRI [60,105-109]. The breadth of these recommendations further underscores the need for a standardized approach of response assessment.

## Summary and Conclusions

Despite advances in treatment, glioblastoma has no cure, and patients with glioblastoma have poor long-term survival. Increased understanding of the tumorigenesis of this disease at the molecular level has led to the identification of VEGF and its related pathways as targets for therapy. As a result, a number of antiangiogenic therapies have been or are currently being evaluated in patients with glioblastoma, alone or in combination with chemotherapy and/or radiotherapy. The most well-established antiangiogenic therapy is bevacizumab; current experience encompasses clinical data from more than 1000 patients treated for glioblastoma. In May 2009, single-agent bevacizumab was approved by the FDA for the treatment of patients with progressive glioblastoma following prior therapy on the basis of an improvement in objective response rate. The BRAIN study that supported this approval also showed a significant improvement in 6-month PFS rate with bevacizumab alone and in combination with irinotecan relative to historical controls [31]. At present, the NCCN guidelines include a recommendation for bevacizumab either with or without chemotherapy as a treatment option for recurrent glioblastoma [4]. The safety and efficacy of cilengitide with chemotherapy has not been reported in the recurrent setting, but single-agent data suggest that combinatorial trials are warranted.

Clinical studies have also demonstrated the feasibility of combining bevacizumab or cilengitide plus radiation with or without concomitant temozolomide for the treatment of patients with newly diagnosed or recurrent glioblastoma. Early data suggest the possibility of novel regimens that improve tumor response without overlapping toxicities, but these findings are preliminary. The incorporation of antiangiogenic agents in frontline therapy, therefore, cannot be recommended at present, except in the context of a clinical trial.

Although the safety and efficacy of combining antiangiogenic agents with chemotherapy has been documented in the recurrent setting, the ideal chemotherapy partner has yet to be identified by prospective, randomized trials. The difficulty of comparing data across trials prohibits any definitive conclusions, and the efficacy signals to date do not provide a clear indication as to which chemotherapy agents or treatment schedules are optimal. Moreover, the scheduling, timing, and dosing of antiangiogenic agents relative to chemotherapy also remains to be defined, and should be a focus of future studies. As the field progresses toward patient-specific approaches, gene expression studies and other correlative analyses are needed to assess the safety and efficacy of antiangiogenic therapies on the basis of the molecular pathophysiology of the disease. Data obtained from ongoing studies should enable clinicians to further optimize treatment for both newly diagnosed and recurrent glioblastoma (Additional file 1, Table S1). Additional information can be found at http://www.ro-journal.com. Alternate treatment strategies for patients with glioblastoma may include the use of an antiangiogenic agent with other targeted agents, such as erlotinib, dasatinib, or cetuximab [110,111]. More research is also needed to establish the most advantageous sequencing for individual components of combination regimens containing antiangiogenic therapies. Antiangiogenic agents are expected to play a significant role in the treatment of glioblastoma in the future, and it is hoped that the consideration of molecular profiling will further improve target selection.

## Competing interests

Dr. Beal acted as a consultant for Hoffman-La Roche at a single meeting. Her institution, Memorial Sloan-Kettering Cancer Center, has received research funding from Genentech, Inc., and Astra Zeneca. Dr. Abrey has been a consultant and on an advisory board for Genentech, Inc. Her institution, Memorial Sloan-Kettering Cancer Center, has received research funding from Genentech, Inc., and Astra Zeneca. Dr. Gutin has been on an advisory board for Genentech, Inc. His institution, Memorial Sloan-Kettering Cancer Center, has received research funding from Genentech and Astra Zeneca.

## Authors' contributions

KB, LEA, and PHG have contributed to the conception of the manuscript and the interpretations of the data contained within, and have been involved in critically revising the manuscript. All authors read and approved the final manuscript.

## Supplementary Material

Additional file 1 **Proposed and ongoing phase II and III trials of antiangiogenic agents in glioma**. Table of proposed and ongoing phase II and phase III trials of select antiangiogenic agents for the treatment of recurrent and newly diagnosed glioma, with study details including NCT numbers, disease setting, primary endpoints, and leading study center. Click here for file
