# Supplementary material for: Antiangiogenic agents in the treatment of recurrent or newly diagnosed glioblastoma: Analysis of single-agent and combined modality approaches
Source: Radiat Oncol. 2011 Jan 7;6:2. doi: 10.1186/1748-717X-6-2 (PMC3025871; doi:10.1186/1748-717X-6-2)
Supplement: Additional file 1 — Proposed and ongoing phase II and III trials of antiangiogenic agents in glioma. Table of proposed and ongoing phase II and phase III trials of select antiangiogenic agents for the treatment of recurrent and newly diagnosed glioma, with study details including NCT numbers, disease setting, primary endpoints, and leading study center. [file 1748-717X-6-2-S1.DOC]

**Additional file 1, Table S1. Proposed and ongoing phase II and III trials of antiangiogenic agents in glioma [114]**

| **Agents**  **(NCT#)** | **Phase** | **Disease setting** | **Primary end point** | **Additional notes** | **Leading study center** |
| --- | --- | --- | --- | --- | --- |
| **Antiangiogenic therapy + chemotherapy** | | | | | |
| Bevacizumab + carmustine (NCT00795665) | II | Relapsed or progressive high-grade glioma | PFS, OS, TTP, response rate, safety |  | University of California, Davis |
| Carmustine wafers then bevacizumab + irinotecan (NCT00735436) | II | Recurrent glioblastoma | 24-week survival | EIAEDs vs non-EIAEDS | Duke University |
| Bevacizumab + enzastaurin (NCT00586508) | II | Recurrent malignant glioma | TTP and safety |  | Multiple |
| Bevacizumab + enzastaurin (NCT00559923) | II | Recurrent malignant glioma | 6-month PFS and safety | Sponsored by the NCI | Multiple |
| Bevacizumab + irinotecan (NCT00921167) | II | Recurrent glioma | PFS (at 6 months and 1 year) |  | Clinical Research Center for Solid Tumor, Korea |
| Low-dose bevacizumab + lomustine vs standard-dose bevacizumab (NCT01067469) | II | Recurrent glioblastoma | PFS | 2-arm study | M.D. Anderson Cancer Center |
| Bevacizumab + temozolomide (NCT01149850) | II | Newly diagnosed glioblastoma or gliosarcoma | OS (at 6 months) | Limited to elderly patients | University of California, Los Angeles |
| Bevacizumab + biweekly temozolomide (NCT00883298) | II | Recurrent glioblastoma or gliosarcoma | 6-month PFS |  | Center for Neurosciences, Tucson |
| Bevacizumab + temozolomide  (NCT01115491) | II | Recurrent glioblastoma | 6-month PFS |  | Multiple |
| Bevacizumab + temozolomide (NCT00590681) | II | Newly diagnosed glioblastoma | Objective response and PFS | After RT and chemotherapy | University of Chicago |
| Bevacizumab + temozolomide + irinotecan (NCT00979017) | II | Unresectable multifocal glioblastoma or gliosarcoma | Response rate |  | Duke University |
| Bevacizumab + vorinostat + temozolomide (NCT00939991) | I/II | Recurrent malignant glioma | 6-month PFS (phase II) |  | Duke University |
| Cilengitide + temozolomide + procarbazine (NCT01124240) | II | MGMT-promoter unmethylated glioblastoma | 12-month PFS |  | Multiple |
| CYT997 + carboplatin (NCT00650949) | I/II | Progressive glioblastoma | 6-month PFS and safety |  | Multiple |
| Vandetanib + carboplatin vs carboplatin (NCT00995007) | II | Recurrent high-grade glioma | Antitumor activity | No EIAEDs; sponsored by the NCI | National Institutes of Health Clinical Center |
| **Antiangiogenic monotherapy** |  |  |  |  |  |
| CT-322 ± irinotecan  (NCT00562419) | II | Recurrent glioblastoma | 6-month PFS and safety |  | Multiple |
| Ramucirumab or IMC-3G3 (NCT00895180) | II | Recurrent glioblastoma | 6-month PFS | Sponsored by the NCI | Multiple |
| Sunitinib  (NCT00535379) | II | Progressive or recurrent glioblastoma | 6-month PFS and median TTP |  | Medical University Innsbruck |
| Sunitinib  (NCT00606008) | II | Recurrent AA or glioblastoma | 6-month PFS |  | H. Lee Moffitt Cancer Center and Research Institute |
| Sunitinib (NCT00923117) | II | Recurrent malignant glioma | Bevacizumab-naive and bevacizumab-exposed patients | Sponsored by the NCI | National Institutes of Health Clinical Center |
| XL184  (NCT00704288) | II | Progressive or recurrent glioblastoma | Objective response rate and safety | Disease in first or second relapse | Multiple |
| **Antiangiogenic therapy and RT** |  |  |  |  |  |
| Concurrent chemoradiation + adjuvant temozolomide ± bevacizumab (NCT00884741) | III | Newly diagnosed glioblastoma | PFS and OS | Sponsored by RTOG and the National Cancer Institute | Multiple |
| RT + temozolomide + bevacizumab or placebo (NCT00943826) | III | Newly diagnosed glioblastoma | OS and PFS |  | Multiple |
| RT + temozolomide → bevacizumab + temozolomide + erlotinib (NCT00525525) | II | Newly diagnosed glioblastoma or gliosarcoma | OS and PFS (at 6 months and 1 year) |  | University of California, San Francisco |
| Radiosurgery + bevacizumab + temozolomide (NCT01086345) | II | Recurrent glioblastoma | OS |  | Case Comprehensive Cancer Center |
| RT + temozolomide → bevacizumab + irinotecan or observation (NCT01022918) | II | Naive unresectable glioblastoma | 6-month non-progressive disease rate | 2-arm study | Centre Georges Francois Leclerc |
| Neoadjuvant temozolomide ± bevacizumab → RT + temozolomide ± bevacizumab → maintenance temozolomide (NCT01102595) | II | Unresectable glioblastoma | Response rate | 2-arm study | Grupo Español de Investigación en Neurooncología |
| Temozolomide + RT + bevacizumab → bevacizumab + temozolomide + topotecan (NCT01004874) | II | Newly diagnosed glioblastoma or gliosarcoma | PFS |  | Duke University |
| Bevacizumab + irinotecan or temozolomide + RT  (NCT00817284) | II | Newly diagnosed glioblastoma | Overall response rate | 2-arm study | Rigshospitalet, Denmark |
| Bevacizumab + irinotecan + RT vs temozolomide + RT→ temozolomide (NCT00967330) | II | Newly diagnosed glioblastoma and a non-methylated MGMT promoter | PFS (at 6 months) | 2-arm study | Multiple |
| Bevacizumab + temozolomide + hypofractionated RT → bevacizumab + temozolomide (NCT00782756) | II | Newly diagnosed malignant glioma | OS |  | Memorial Sloan-Kettering Cancer Center |
| Cediranib or placebo → RT + temozolomide with cediranib or placebo→ temozolomide + cediranib or placebo (NCT01062425) | II | Newly diagnosed glioblastoma | 6-month PFS | 2-arm study | Multiple |
| Cediranib + temozolomide + RT (NCT00662506) | I/II | Newly diagnosed glioblastoma | PFS and safety |  | Massachusetts General Hospital |
| Temozolomide + RT ± cilengitide (NCT00689221) | III | Newly diagnosed glioblastoma and methylated gene promoter status | OS |  | Multiple |
| Temozolomide + RT + cilengitide or cetuximab (NCT01044225) | II | Newly diagnosed MGMT-promoter unmethylated glioblastoma | 1-year OS | 2-arm noncomparative study | Universitair Ziekenhuis Brussel |
| Temozolomide + RT ± cilengitide (NCT00813943) | II | Newly diagnosed glioblastoma and unmethylated gene promoter status | OS | 3-arm study | Multiple |
| Temozolomide + RT ± sorafenib → temozolomide ± sorafenib (NCT00734526) | I/II | Newly diagnosed glioblastoma or gliosarcoma | TTP (phase II) | 4-arm study | M.D. Anderson Cancer Center |
| Sunitinib → RT + sunitinib → sunitinib (NCT01100177) | II | Newly diagnosed glioblastoma | Objective response rate |  | Grupo Español de Investigación en Neurooncología |
| RT + temozolomide ± vandetanib (NCT00441142) | I/II | Newly diagnosed glioblastoma or gliosarcoma | OS (phase II) | 2-arm study | Dana-Farber Cancer Institute |

Abbreviations: AA = anaplastic astrocytoma; EIAEDs = enzyme-inducing antiepileptic drugs; MGMT = O(6)-methylguanine-DNA methyltransferase; NCI = National Cancer Institute; OS = overall survival; PFS = progression-free survival; RT = radiotherapy; RTOG = Radiation Therapy Oncology Group; TTP = time to progression; VEGF = vascular endothelial growth factor. Search term: glioblastoma. Study categorization: Open.
